# Enhanced Radiation Sensitivity of Human Papillomavirus-Driven Head and Neck Cancer: Focus on Immunological Aspects

**DOI:** 10.3389/fimmu.2019.02831

**Published:** 2019-12-03

**Authors:** Mine Özcan-Wahlbrink, Christoph Schifflers, Angelika B. Riemer

**Affiliations:** ^1^Immunotherapy and Immunoprevention, German Cancer Research Center (DKFZ), Heidelberg, Germany; ^2^Molecular Vaccine Design, German Center for Infection Research, Partner Site Heidelberg, Heidelberg, Germany; ^3^Cell Biology Research Unit (URBC)—Namur Research Institute for Life Sciences (NARILIS), University of Namur, Namur, Belgium

**Keywords:** human papillomavirus (HPV), head and neck squamous cell carcinoma (HNSCC), HPV-driven HNSCC, oropharyngeal squamous cell carcinoma (OPSCC), radiation sensitivity, immune response

## Abstract

Head and neck squamous cell carcinomas (HNSCC), emerging in the mucosa of the upper aerodigestive tract, are associated with either the classical risk factors, tobacco and alcohol consumption, or with infections with high-risk types of the human papillomavirus (HPV). Depending on the involvement of HPV, HNSCC follow different pathways of carcinogenesis and show distinct clinical presentations regarding survival, prognosis and treatment response. For instance, HPV-driven HNSCC exhibit an enhanced radiation response compared to their typically radioresistant HPV-negative counterparts. Although radiosensitivity of HNSCC has been studied by many research groups, the major causes for the difference in radiation responses between HPV-driven and HPV-negative HNSCC are still an open question. In this mini review, we discuss the reported cellular and immunological factors involved in the enhanced radiation response in HPV-driven HNSCC, focusing on the vital role of the immune response in the outcome of HNSCC radiotherapy.

## Introduction

**Figure 1:**
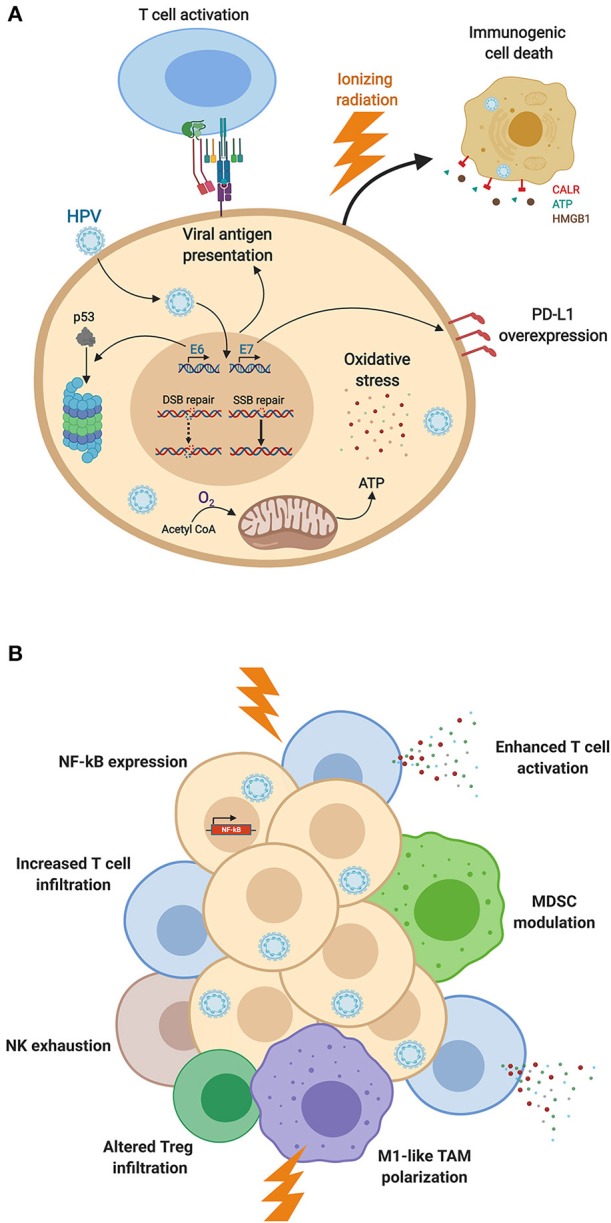
HPV-induced modifications of **(A)** cancer cell biology and **(B)** immune responses, impacting the radiation response. **(A)** Expression of HPV-associated proteins induces adaptations of cellular biology, including DNA repair dysfunction, proteasomal degradation of p53 altering cell cycle distribution, E7-induced PD-L1 expression, HPV-mediated oxidative stress, and viral antigen presentation. These cellular modifications as well as mitochondrial oxidative phosphorylation enhance cancer cell sensitivity to ionizing radiation and promote immunogenic cell death. **(B)** HPV-mediated NF-kB activation, T cell infiltration and activation, and M1-like TAM polarization are enhanced by radiation, promoting anti-cancer immunity after irradiation of HPV-driven HNSCC. HPV-associated MDSC modulation as well as NK cell exhaustion offer additional therapeutic targets to boost anti-tumor responses (Figure created with BioRender.com).

Head and neck squamous cell carcinomas (HNSCC) associated with high-risk human papillomavirus (HPV) infections have emerged as an independent subgroup of HNSCC in recent years (1). It has further been found that it is important to differentiate HPV-positive HNSCC into merely HPV DNA-positive tumors, which however behave like HPV-negative ones, and tumors that express HPV RNA and proteins. The latter are termed “HPV-driven” and show a different biology (2). HPV-driven tumors represent about 25% of all HNSCC, and arise in specific sites in the oropharynx, namely the tonsils and base of the tongue. At these sites, they constitute up to 80% of all squamous cell carcinomas (3), which has resulted in oropharyngeal squamous cell carcinomas (OPSCC) being treated separately from other HNSCC in the new WHO Classification of Head and Neck Tumors (4). HPV-driven HNSCC are characterized by their significant better prognosis and survival advantage over HPV-negative HNSCC (5–7). Also, HPV-driven HNSCC have been observed to have a superior radiation response compared to their typically radioresistant HPV-negative counterparts (8–11). Enhanced survival and better radiation response of HPV-driven HNSCC have been assessed and reviewed by many papers; however, the major causes are still discussed, as many conflicting results have been reported (9, 12–17) (reviewed below). A tumor's response to radiotherapy is commonly determined by the so-called *6 Rs of Radiobiology* including DNA **R**epair, cell cycle **R**edistribution, tumor **R**eoxygenation, **R**epopulation, cancer cell intrinsic **R**adiosensitivity and **R**eactivation of the anti-tumor immune response (18, 19). The particularly enhanced radiation response of HPV-driven HNSCC might be related to one or more of the above-mentioned factors, especially considering that radiation responses are known to be strongly determined by the cell intrinsic ability to sense DNA damage, trigger a DNA damage response (DDR) and mediate DNA repair (20). In this mini review, we cover the cellular as well as the immunological characteristics of HPV-driven vs. HPV-negative HNSCC that may result in different radiation responses (Figure 1).

## Cellular Mechanisms

Numerous research groups have investigated the cellular basis of the observed differential radiosensitivity of HPV-driven and HPV-negative HNSCC, hypothesizing that viral proteins may affect the cellular radiation response. Indeed, recent work has shown that HPV inhibits the anti-viral cGAS-STING pathway, influences the cellular DNA repair machinery, alters cell cycle distribution, affects apoptosis as well as DNA replication and mediates unique kinetics of hypoxia during radiotherapy (12–16, 21–25). By analyzing cancerous and healthy tissue, Foy et al. established a radioresistance score based on the expression of 13 genes, RadR, that can potentially be utilized to predict radioresistance or radiosensitivity and thus the outcome of radiotherapy (26). The RadR score was correlated with genes in seven essential pathways: TGFβ signaling, DNA repair, angiogenesis, unfolded protein response, E2F targets, Myc targets and epithelial to mesenchymal transition. Although HPV-negative HNSCC were shown to be heterogeneous in the RadR score, HPV-driven HNSCCs had significantly lower scores, in line with the known superior radiation response of HPV-driven HNSCC (26). In the following, we discuss those pathways that are differentially regulated between HPV-driven and HPV-negative HNSCC, and highlight the most critical components for radiosensitivity.

Ionizing radiation eradicates cancer cells by inducing DNA damage, either directly or indirectly by the formation of free radicals. Therefore, the **DNA repair system** is crucial for the sensitivity of cancer cells toward irradiation. Liu et al. reported that abrogation of TGFβ signaling by HPV results in DNA repair deficiencies, which consequently cause elevated radiosensitivity in HPV-driven HNSCC (27). Furthermore, several groups revealed that HPV-driven HNSCC cells have DNA double-strand break (DSB) repair defects (12, 16, 28), specifically due to affected non-homologous end joining (29). Interestingly, HPV-driven HNSCC cancer cells overexpress proteins involved in base excision repair and single-strand break (SSB) repair (16). Although defects in the DSB repair system would contribute to enhanced radiosensitivity, it is unknown how increased SSB repair would affect cancer cells, particularly considering that the HPV oncoprotein E6 was shown to bind XRCC1, a factor required for SSB repair (30). On the other hand, it was recently found in patients that radioresistance of HPV-negative HNSCC was associated with overexpression of BAP-1, potentially via the promotion of homologous-recombination-mediated DNA repair and histone H2A deubiquitination (31). Then again, HPV promotes radiosensitivity of HNSCC by suppressing non-homologous end joining and promoting error-prone microhomology-mediated end-joining by the expression of the oncoprotein E7 (32). Lastly, it was shown that p16^INK4a^ overexpression, the most established surrogate marker for diagnosing HPV involvement, suppresses homologous recombination-dependent DNA repair through suppression of RAD51 foci formation (33) and decreased expression of TRIP12 (34). Since DNA repair is particularly important after radiotherapy to restore genomic integrity, HPV sensitizes HNSCC to irradiation by impairment of error-free as well as promotion of error-prone DNA repair mechanisms.

Differential **regulation of cell cycle** components has also been shown to affect the radiation response. For instance, Lohavanichbutr et al. discovered that the differential expression of Retinoblastoma-binding protein 4 and cyclin D1 genes (*RBBP4* and *CCND1*) was associated with radiosensitivity in HPV-driven OPSCC (24). Sepiashvili et al. reported that differential regulation of several target genes of AP-1, such as cyclin-dependent kinase inhibitor 2A *(CDKN2A)* and *TP53*, leads to radioresistance in HPV-negative OPSCC (25). A major cell cycle-related cause of the enhanced radiation response, as thoroughly discussed by many papers, stems from the presence or absence of functional **p53** protein. While it has been demonstrated that *TP53* is commonly mutated in HPV-negative HNSCC but not in HPV-driven HNSCC (35), the HPV E6 oncoprotein induces proteasomal degradation of p53 leading to low amounts of the functional form of this tumor suppressor in HPV-driven tumors (36). Whether these low amounts of functional p53 affect the radiosensitivity of HPV-driven HNSCC however remains questionable. Indeed, despite the fact that complete knock-down of p53 was shown to induce radioresistance in HPV-driven HNSCC (37), Seltzsam et al. recently showed that radiation-induced p53 pathway activation after functional restoration of p53 by inhibition of proteasomal degradation does not sensitize HNSCC cells to irradiation (38). Furthermore, Pang et al. reported p53-independent radiation-induced death of HPV-driven HNSCC cells (39). Therefore, even if p53 plays an important role in the radiation response, it cannot be the main and mere factor of HPV-driven HNSCC radiosensitivity.

Differential **regulation of the tumor metabolism** has been shown to enhance HPV-driven HNSCC radiosensitivity. Jung et al. demonstrated that HPV-negative HNSCC display high rates of glycolysis in comparison to HPV-driven HNSCC, whose energy supply is predominantly fueled by mitochondrial oxidative phosphorylation. Additionally, in contrast to their HPV-driven counterparts, HPV-negative HNSCC were characterized by high expression levels of HIF1α, promoting cancer cell resistance and aggressiveness (40). Pharmacological manipulation to reverse the glycolytic phenotype in HPV-negative HNSCC cells resulted in elevated radiosensitivity (40). Supporting this data, the effect of hypoxia has been evaluated in many papers (41–43); however, these studies surmise that hypoxia may only partially play a role in radioresistance of HPV-negative HNSCC, and no effect of hypoxia on radiosensitivity was detected for HPV-driven HNSCC. Another important metabolic factor determining radiosensitivity is oxidative stress, as induced by ionizing radiation. Several HPV proteins have been shown to increase levels of reactive oxygen and nitrogen species in HNSCC cells and to alter the expression of antioxidant enzymes leading to impaired oxidative stress reduction (44). Therefore, HPV-driven HNSCC metabolism plays an important role in the increased radiosensitivity, potentially through a synergistic action between HPV-induced and radiation-induced oxidative stress.

## Immune Responses

In addition to differences in DNA repair, cell cycle control and tumor metabolism, the importance of an intact immune system for the radiation response was highlighted by Spanos et al. They examined the effect of radiotherapy in HPV-driven and HPV-negative HNSCC *in vitro* as well as in immune-competent and immune-incompetent mice. They showed that HPV-driven tumors were more sensitive to irradiation in immune-competent mice but not in mice lacking an adaptive immune system. These results suggest that an intact immune system plays a crucial role in the radiosensitivity of HPV-driven HNSCC compared to HPV-negative HNSCC (45).

There are many publications correlating specific immune parameters in HPV-driven HNSCC patients with better disease-free and overall survival (46–49). This is most likely due to immune responses triggered by HPV infection, which may be further enhanced by radiotherapy (20). It is well-documented that radiation promotes anti-cancer immune responses in various types of cancer, by favoring immunogenicity via immunogenic cell death, increased antigen presentation, promoting inflammation, dendritic cell maturation, and T cell activation (50–55). Therefore, the impact of HPV on any of these immune-related mechanisms could contribute to the higher treatment response and improved prognosis of HPV-driven vs. HPV-negative HNSCC. So far, none of the published studies could however establish a direct relationship between the immune response in HPV-driven HNSCC and their increased radiosensitivity. Thus, in the following we discuss the known immunological aspects of HPV-driven HNSCC, which are potentially modifying tumor radiosensitivity in comparison to HPV-negative HNSCC.

It has been shown that HPV-driven OPSCC present viral antigens that elicit HPV-oncoprotein-specific antibody as well as T cell responses. These responses are believed to be due to the tight anatomical proximity of mucosa and immune tissue in the oropharynx (tonsils) (56, 57). Especially T cells responses are thought to participate in tumor rejection and long-term immune surveillance. In addition, radiation has been described to promote T cell-mediated anti-tumor immunity (55). Several groups examined **tumor infiltrating lymphocytes (TILs)** in HPV-negative and HPV-driven HNSCC (58), specifically in HPV-driven OPSCC (57, 59–62), and demonstrated that compared to HPV-negative HNSCC, HPV-driven tumors were infiltrated by significantly more immune cells. A recent study further showed that HPV-driven OPSCC have stronger immune cell infiltration than HPV-driven HNSCC at other sites (63). Analysis of CD8^+^ cytotoxic TILs showed that HPV-driven tumors displayed higher levels of activated CD8^+^ T cell infiltration with elevated effector cytokine expression (58), despite the fact that Liu et al. reported HPV-associated E7 expression directly favoring T cell dysfunction by PD-L1 overexpression (64). The correlation of CD8^+^ T cell infiltration with overall survival in HPV-driven and in HPV-negative HNSCC demonstrated that independent of the HPV status, higher cytotoxic T cell infiltration is associated with increased overall survival (57, 59, 61). These results are in line with recent *in silico* studies analyzing The Cancer Genome Atlas (TCGA) data, that showed a higher level of T cell signatures in HPV-positive compared to virus-negative tumors (58, 63, 65–67), and especially that an immune response signature is associated with a favorable prognosis in patients with HPV-driven HNSCC (58, 63, 65, 66). Furthermore, Hess et al. established an immune signature risk score (ISRS), based on the expression on 13 genes, robustly distinguishing patients with either a more favorable overall survival prognosis or a less favorable prognosis. Even though HPV-driven HNSCC patients were present in both subgroups, they clearly accumulated in the group with better overall survival (67). These results highlight that inflammation and T cell responses as promoted by HPV as well as irradiation might contribute to an improved response to radiotherapy in HPV-driven HNSCC.

On the other hand, some immune cell types are frequently described to be pro-tumorigenic and immunosuppressive. The most prominent examples are **tumor associated macrophages (TAMs) and regulatory T cells (Tregs)**. Lee et al. defined the number of CD68^+^ TAMs and the distribution of Tregs as negative factors that determine the outcome of concurrent chemoradiotherapy in HPV-driven tonsillar cancer (68). Nevertheless, it should be considered that TAMs are highly plastic cells that can act pro- or anti-inflammatory. In this context, Chen et al. discovered a predominantly M1-like proinflammatory phenotype in HPV-driven cancer (65, 69), which favors an enhanced radiation response (69). However, the phenotype of TAMs and therefore their role in long-term treatment response is known to be affected by various anti-cancer therapies including radiotherapy, as reviewed in further detail by Genard et al. (70). Treg infiltration in HPV-driven tumors was analyzed by Mandal et al. (58), Punt et al. (59), and Bron et al. (71). Surprisingly, all these studies reported a higher Treg infiltration than in HPV-negative HNSCC, and a correlation of Treg infiltration with good prognosis. Recently, Santegoets et al. focused the analysis on Tbet-positive Tregs, which also were found to be increased in HPV-driven OPSCC, and correlated with improved survival (72). The authors argue that these counterintuitive findings most likely reflect Treg recruitment by the presence of a strong ongoing (effector T cell based) protective immune response, with the net effect being anti-tumorigenic. Similarly, PD-1 expressing tumor-infiltrating T cells were found at higher frequencies in HPV-driven HNSCC, and were positively correlated with a favorable outcome (73, 74). This again most likely reflects an ongoing protective immune response, with PD-1 representing an activation rather than an exhaustion marker.

Additionally, Mandal et al. characterized another cell type to have an “exhausted” phenotype: both HPV-driven and HPV-negative HNSCC were infiltrated with **CD56^dim^ NK cells** expressing the inhibitory killer cell immunoglobulin-like (KIR) receptor (58). Despite intrinsically inducing unfavorable immune responses, an exhausted or immunosuppressive phenotype of defined cell subsets could be targeted and influenced by using immunomodulators such as anti-PD-1/PD-L1 antibodies, anti-CTLA-4 antibodies and anti-KIR antibodies. Indeed, the KEYNOTE-012 trial, testing the anti-PD-1 antibody pembrolizumab in PD-1-positive HNSCC patients, reported a higher rate of overall responses in patients with HPV-positive tumors (25%) as compared to HPV-negative ones (14%) (74). These results suggest that the combination of immune-checkpoint blockage with radiotherapy, which is reported to promote immunogenicity of various types of cancer cells (51), might harbor important potential for the treatment of head and neck cancer, particularly HPV-driven HNSCC.

Lastly, **CD11b^+^LIN^−^HLA-DR^−^CD33^+^ myeloid-derived suppressor cells (MDSCs)** were also defined to be a pivotal cell population in HPV-driven HNSCC that moderates inflammatory responses and immune suppression (75). In order to modulate the immunosuppressive effect of MDSCs in HNSCC, Jayaraman et al. treated the MDSCs with TGFβ-containing conditioned medium. As a result, it was observed that TGFβ-MDSCs exhibited a novel immunostimulatory phenotype with enhanced antigen presenting capability and no inhibitory effect on T cell proliferation. Increased Fas-L expression by TGFβ-MDSCs led to killing of HPV-driven HNSCC cells. In addition, combination of radiotherapy and intratumoral injection of TGFβ-MDSCs augmented MHC class I expression and promoted the tumor infiltration of HPV E7 tetramer-positive CD8^+^ T cells, resulting in clearance of established tumors and long-term survival in mice (76). The authors also observed that in parallel to the mouse TGFβ-MDSCs, human TGFβ-MDSCs lost their immunosuppressive activity and gained tumor killing characteristics (76). As a result, the immunosuppressive effect of MDSCs may also be manipulated in favor of anti-tumor immune responses, which could affect radiosensitivity.

Apart from immune cells, another immune related factor that is essential in anti-tumor immune responses is MHC class I expression by tumor cells. The effect of MHC class I expression on clinical outcome in HPV-driven HNSCC has been studied only by a few research groups. Interestingly, low MHC class I (HLA-A, B, C) expression of HPV-driven tonsillar squamous cell carcinoma was significantly associated with a favorable clinical outcome (77). This unexpected correlation was further supported by another paper from the same group that correlates absence of HLA class I expression in HPV-driven HNSCC with high survival (78) and by a study by Tertipis et al., claiming that absence of HLA-A^*^02 correlated with better disease-free survival in HPV-driven tonsillar and base of tongue cancer (79). As described by Wagner et al., the improved prognosis of HPV-driven HNSCC despite decreased HLA class I expression might be mediated by increased NK cell cytotoxicity (80). Nevertheless, the findings that describe a correlation between low HLA class I expression and better survival are surprising and need further investigation with larger patient cohorts.

## Conclusion and Outlook

Considering all the factors enhancing the radiation response that have been reviewed above and in the light of the research certifying that HPV-positivity predicts a better outcome (8, 10, 11), we conclude that the cellular factors DNA repair, cell cycle control, and tumor metabolism partially mediate the superior radiation response of HPV-driven HNSCC. However, high immunogenicity of HPV-driven tumors, further enhanced by radiotherapy, constitutes another main factor in radiosensitivity of HPV-driven tumors. Hence, in order to predict the radiation response for HNSCC patients, it is paramount to characterize the immunologically active subtypes of HNSCC. Even if HPV-driven HNSCC tumors are postulated to have an “active immune response” in general (81) and to promote an inflammatory environment by co-activation of classic and alternative NF-κB pathways (82), two distinct HPV-driven HNSCC subtypes have been defined based on gene expression-based consensus clustering that might explain the significant heterogeneity in clinical behavior in HPV-driven HNSCC (83, 84). In this classification, an “inflamed/mesenchymal” subtype was characterized by expression of immune response genes such as *CD8, ICOS* and *HLA-DRA*; decreased expression of epithelial markers and upregulation of mesenchymal markers (suggesting an epithelial-mesenchymal transition signature). The “classical” subtype was represented by enrichment of components of the polyamine degradation pathway, related to an even higher proliferation rate. Associated with HPV infection, both subtypes displayed high cell cycle-related activities (84). The authors revealed that HPV-driven HNSCC have different subgroups, some of which are less immunogenic or lack immune-related markers. A comprehensive classification of HNSCC types based on immune phenotype was recently established by Chen et al. (49). This study describes a new molecular immune phenotyping of HNSCC, called “immune class” depending on the presence of immune cell subsets, cytolytic activity, immune metagenes and enrichment of a 6-gene interferon signature. Application of this phenotyping defined a non-immune class and two groups of immune class. The first of these was “active immune class,” described by enrichment of B cells, M1-like macrophages, cytolytic activity, high numbers of tumor TILs and high HPV infection; it was found to be correlated with a favorable prognosis, better overall and disease-free survival. Of the HNSCC tumors with known HPV status (by p16 immunohistochemistry), 63% assigned to the “active immune class” were HPV-positive. On the other hand, the “exhausted immune class” was characterized by a more “exhausted phenotype” and had tumor-promoting activated stroma, activated TGFβ and Wnt signaling, markers of M2-like TAM polarization and poor survival. Only 13% of “exhausted immune class” HNSCC with known HPV status were HPV-positive. When comparing within the HPV-positive tumors, 67% fell into the “active” category and only 5% into “exhausted” (49).

In summary, characterization of HNSCCs according to their immune-related markers, independent from HPV status, will most likely contribute to tailor therapies more efficiently in the future, and help understand the difference in radiotherapy response between HPV-driven and HPV-negative HNSCC.

## Author Contributions

All authors listed have made a substantial, direct and intellectual contribution to the work, and approved it for publication.

### Conflict of Interest

The authors declare that the research was conducted in the absence of any commercial or financial relationships that could be construed as a potential conflict of interest.
